# Changes in Core Temperature Following Administration of FC5, a Blood–Brain Carrier Fused with Neurotensin in Mice, Rats and Non-Human Primates

**DOI:** 10.3390/cells15191770

**Published:** 2026-09-29

**Authors:** Alvaro Yogi, Etienne Lessard, Eric Brunette, Christie E. Delaney, Arsalan S. Haqqani, Anna Robotham, Wael Alata, Maria J. Moreno, Danica B. Stanimirovic

**Affiliations:** 1 Human Health Therapeutics Research Centre, National Research Council Canada, Ottawa, ON K1A 0R6, Canada; etienne.lessard@cnrc-nrc.gc.ca (E.L.); eric.brunette@nrc-cnrc.gc.ca (E.B.); arsalan.haqqani@nrc-cnrc.gc.ca (A.S.H.); anna.robotham@nrc-cnrc.gc.ca (A.R.); maria.moreno@nrc-cnrc.gc.ca (M.J.M.);; 2 Biology Program, Division of Science, New York University Abu Dhabi, Saadiyat Island Campus, Abu Dhabi P.O. Box 129188, United Arab Emirates; walata@lacitec.on.ca

**Keywords:** blood–brain barrier, receptor-mediated transcytosis, single domain antibody, FC5, non-human primate, core temperature, neurotensin

## Abstract

Development of therapies and pharmacological management of central nervous system diseases is significantly hampered by the presence of the highly selective blood–brain barrier (BBB). Receptor-mediated transcytosis (RMT) has proven to be a robust mechanism for delivering a wide variety of therapeutic modalities across the BBB. The present study describes the use of a neurotensin-based pharmacodynamic model to evaluate the brain penetrating capacity of FC5, a well-characterized RMT shuttle. Here we produce fusion molecules of FC5 with human Fc and neurotensin and show that intact but not C-terminally clipped neurotensin proteins containing either FC5 (FC5Fc-neurotensin) or a non-BBB-crossing control (A20.1Fc-neurotensin) retain the ability to activate neurotensin receptor type 1. In in vivo studies, intravenous administration of FC5Fc-neurotensin, but not neurotensin alone or A20.1Fc-neurotensin, induced a drop in the core body temperature in both rats and mice. We further extended these findings to non-human primates, demonstrating the ability of FC5 to cross the BBB in this species. Collectively, our results further support the potential of FC5 in delivering payloads across the BBB. The neurotensin model of hypothermia is also validated as a pharmacodynamic approach for assessing brain delivery, albeit with some necessary considerations into the molecular characterization of fusion constructs before in vivo evaluation.

## 1. Introduction

Despite significant advances in the understanding of the pathophysiology of neurodegenerative diseases, current treatment options remain extremely limited. One of the major reasons is the restricted drug delivery to the brain due to the presence of a highly selective blood–brain barrier (BBB). Whereas the BBB preserves neural homeostasis and the central nervous system (CNS) integrity by limiting exposure to potentially harmful circulating agents, it simultaneously restricts the entry of most therapeutic agents [1]. In fact, most small-molecule drugs, as well as biologic therapeutics such as peptides, proteins, antibodies and nucleic acid-based therapies, do not efficiently cross the BBB, significantly hampering the development of therapies and pharmacological management of CNS diseases [2].

Receptor-mediated transcytosis (RMT) has emerged as a robust mechanism for delivering a wide variety of therapeutic modalities across the BBB, with multiple receptor systems exploitable for this purpose [3]. To date, several receptors have been demonstrated to enhance drug delivery across the BBB, including transferrin (Tf) receptor (TfR), insulin receptor (IR), low-density lipoprotein receptor-related protein 1 (LRP1), insulin-like growth factor receptors (IGF1R) and transmembrane protein 30A (TMEM30A/CDC50A) [4,5,6,7,8]. The discovery of a new BBB carrier framework involves target identification, molecular optimization and the in vitro demonstration of transmigration. Conventional, in vitro BBB models include immortalized brain endothelial cell lines and primary brain endothelial cells of either human or animal origin [9]. More recently, models based on human-induced pluripotent stem cell (iPSC)-derived brain endothelial cells have shown to be promising alternatives, generating tight endothelial-like monolayers with low paracellular permeability [10,11]. Physiologically relevant microfluidic dynamic BBB models may provide additional tools for identifying and characterizing novel BBB carriers, although further validation of these systems is still required [12]. Nonetheless, the complex anatomy and physiology of the CNS limit the predictive value of in vitro BBB models, underscoring the need for complementary in vivo pharmacokinetic and pharmacodynamic approaches to confirm functional brain exposure.

One important hurdle in the BBB carrier development is demonstrating that a candidate can not only cross the BBB but also deliver a pharmacologically active payload into the CNS at sufficient concentrations to induce a pharmacodynamic response. Animal models in which a physiological readout is known to be mediated by central mechanisms are particularly useful in determining whether a carrier can facilitate brain penetration following peripheral administration. We have previously used centrally active analgesic peptides such as galanin and evaluated their ability to elicit a pharmacological response (reversal of hyperalgesia) in an inflammation-induced thermal hyperalgesia pain model [4,13]. More recently, we, and others, have also used core body temperature as a readout to assess brain delivery based on the hypothermic properties of neurotensin (NT) [4,14,15,16,17,18,19]. NT is a 13 amino acid peptide (amino acid sequence Glu-Leu-Tyr-Glu-Asn-Lys-Pro-Arg-Arg-Pro-Tyr-Ile-Leu-OH) first identified from bovine hypothalamic extracts [20]. NT mediates its effects through three receptors, the high-affinity NTS1, the low-affinity NTS2 and NTS3. NTS1, a G-protein-coupled receptor, is the most studied NT receptor and is expressed in neurons and broadly distributed in the CNS [21]. When injected directly into the brain, NT induces rapid and transient hypothermia in rodents that is mediated through hypothalamic NTS1-dependent pathways [22]. Given that NT has a very low BBB permeability and since the CNS effects of NT are limited to central local release or central administration, the fusion of NT to BBB-targeting antibodies has thus been employed to screen the CNS penetration of potential BBB carriers [4,14,15,16,17,18,19,23].

Limited cross-species translatability also contributes substantially to the high rate of failures encountered when therapeutics advance from pre-clinical studies to clinical trials, exacerbating the already high attrition rate in CNS drug discovery [24]. These disappointing results can arise from important interspecies differences in biological complexity, physiology and regulatory mechanisms. Accordingly, demonstrating the activity of BBB carriers across multiple species is an important step toward establishing their translational potential for brain delivery applications.

The present study describes the use of a neurotensin-based pharmacodynamic model to determine the brain penetrating capacity of FC5, a known RMT carrier. FC5 is a single-domain antibody (VHH) that binds to TMEM30A. It was originally selected by functional panning of phage-display llama VHH library for their ability to internalize into human brain endothelial cells (BEC) and was shown to transmigrate across the BBB both in vitro and in vivo [6,25]. Here we demonstrate that the fusion of FC5 with human Fc and NT (FC5Fc-neurotensin), but not NT alone or NT fused with a non-BBB-crossing VHH (A20.1Fc-neurotensin), induces a reduction in the core body temperature following the intravenous injection in rats and mice. Importantly, we extend previous applications of this pharmacodynamic model by demonstrating the ability of FC5 in crossing the BBB in non-human primates (NHPs). Taken together, our results support the use of the NT-induced hypothermia model as a functional readout to determine permeability of BBB carriers across multiple species, while providing important considerations for the characterization of BBB-targeting constructs prior to in vivo administration.

## 2. Materials and Methods

### 2.1. Expression and Purification of VHH FC5, A20.1 and Fusions with Fc Domain and Neurotensin

FC5, the llama single domain antibody used in this study was selected by functional panning of naive llama VHH phage-display library for their ability to internalize into human brain endothelial cells (BEC) and to cross the BBB in vitro [6,25]. A20.1, used here as a non-BBB crosser control, was raised following immunization against *Clostridium difficile* toxin A and does not bind to any known target in mammalian cells [25]. DNA–encoding A20.1Fc, A20.1-Neurotensin and FC5Fc-Neurotensin were synthesized using Genescript. The neurotensin fusion sequences include a linker (amino acid sequence GGGSGGGGS). Constructs were expressed either in transiently transfected Chinese hamster ovary cells (CHO-3E7) or stable CHO cell pools (CHOBRI^TM^). The culture medium was harvested 7 days post-transfection via centrifugation and clarified using 0.2 µm filter bottles (Millipore Stericup, MilliporeSigma, Burlington, VT, USA). Fusion proteins were purified from clarified and filtered culture medium on recombinant Protein A Sepharose Fast Flow (Cytiva, Wilmington, DE, USA) at 10 mg mAb/mL resin, then concentrated in a Vivaspin 5 kDa (Vivaproducts, Littleton, MA, USA), and chromatographed on a Superdex-200 (2.6 × 87 cm) column (Cytiva, Wilmington, DE, USA) to eliminate protein aggregates. Constructs were then sterile-filtered by passing through a Millex GP (MilliporeSigma, Burlington, VT, USA) filter unit (0.22 µm) and aliquoted. The purity of the protein was verified using SDS-PAGE and they were stored at −80 °C. Endotoxin levels were determined using the Endosafe system (Charles River, Charleston, SC, USA). Samples were subjected to SDS-PAGE on 4–12% Bis-Tris NuPAGE gradient gel (Invitrogen, Carlsbad, CA, USA). Non-reduced samples were treated with 5 mM N-ethyl maleimide for 5 min at room temperature, diluted with non-reducing sample buffer, heated at 95 °C for 2 min, and electrophoresed. Reduced samples were treated with 2% 2-mercaptoethanol buffer and heated as above. Size-exclusion chromatography (SEC) was carried out on a BioSep s3000 SEC column (300 × 7.8 mm; Phenomenex, Torrance, CA, USA) in phosphate-buffered saline (PBS), pH 7.2, and 150 mM NaCl) at a flow rate of 0.6 mL/min using a Waters Alliance instrument (Millipore, Milford, MA, USA). In addition to ultraviolet detection, the eluent was monitored with an Optilab Rex refractive index detector (Wyatt, Santa Barbara, CA, USA). Light scattering was monitored using a Treos MiniDawn detector (Wyatt, Santa Barbara, CA, USA). Molecular mass of each complex was determined using the Wyatt Astra 8 software.

### 2.2. Intact Protein LC-ESI-MS

The identity and purity of the samples were assessed by intact protein liquid chromatography–electrospray ionization-mass spectrometry (LC-ESI-MS) analysis. First, protein samples at 0.5 mg/mL in 50 mM Tris-HCl pH 7 were deglycosylated with 0.1 U PNGaseF (MilliporeSigma, Burlington, VT, USA) per μg of antibody overnight at 37 °C. The deglycosylated samples were injected using an Agilent 1100 HPLC system onto a 2.1 × 30 mm Poros R2 reverse phase column (Applied Biosystems, Waltham, MA, USA). Proteins were desalted using a 3 mL/min 0.1% formic acid aq/acetonitrile (degassed) linear gradient (10–75% acetonitrile over 3 min). The column and solvents were heated to ~80 °C to improve protein peak shape. The column was coupled via an Ion Max electrospray source to an LTQ-Orbitrap XL mass spectrometer (ThermoFisher Waltham, MA, USA) and spectra were acquired with cone voltage 0 V, scan range 400–2000 *m*/*z*, and FT resolution 15,000. The mass spectra acquired for each protein were summed and the ion envelopes were deconvoluted into a molecular weight profile using the MaxEnt 1 module of MassLynx (Waters, Milford, MA, USA).

### 2.3. NTS1 Receptor Activation

A receptor functional assay was carried out using the PathHunter eXpress NTR1 kit (DiscoverX, Fremont, CA, USA). Briefly, engineered CHO-K1 cells expressing a (Pro-Link or PK)-tagged NTS1 and an enzyme acceptor (EA)-tagged SH2 domain were used. Cells were thawed and plated in a 384-well white-wall clear-bottom plate (Greiner, Monroe, LA, USA) at 20,000 cells/well for 24 h at 37 °C in 5% CO_2_ following the manufacturer’s instructions. Cells were then treated with neurotensin (MilliporeSigma, Burlington, VT, USA) or FC5Fc-neurotensin and A20.1Fc-neurotensin fusion proteins. The chemiluminescent substrate was added and cells were incubated at RT for 60 min. The resulting luminescence was measured using the CLARIOstar plate reader (BMG LABTECH, Ortenberg, Germany) and concentration–response curves were generated using nonlinear regression analysis (GraphPad Prism 11 Software, San Diego, CA, USA).

### 2.4. Animals

Male Wistar rats (weight range, 150–200 g) male BALB/c (4-week-old) and male CD-1 mice (22–30 g) were purchased from Charles River Laboratories, Inc. (Montreal, QC, Canada). Animals were housed in a 12 h light–dark cycle at a temperature of 24 °C, relative humidity of 50 ± 5% and were allowed free access to food and water. Male Cynomolgus monkeys (2.75 to 4 years old; 2.9–3.6 kg, Charles River Laboratories, Laval, QC, Canada) were used in this study. The animals were maintained in constant environment conditions (temperature 21 ± 3 °C; humidity 30–70%; 10–15 air changes per h; 12 h light–dark cycle). A standard certified commercial primate chow (Certified Hi-Fiber Primate Diet 7195CTM; Harlan Teklad, Madison, WI, USA) and fresh fruits were available to each monkey twice daily, and water was available ad libitum. All experimental procedures were conducted in accordance with the Canadian Council on Animal Care (CCAC) and the procedures were reviewed and approved by respective Animal Care Committees, and conducted as per standard operating procedures in place.

### 2.5. Measurement of Core Temperature

All animals were allowed to acclimatize to the facility for a period of at least 5 days prior to any procedure. Before surgery, animals were injected with sustained release buprenorphine (1.2 mg/kg) subcutaneously for analgesia. Temperature data loggers were implanted in the peritoneal cavity of rats (DST micro-T, Star-Oddi, Iceland) and mice (DST nano-T, Star-Oddi, Iceland) under isoflurane anesthesia. Animals were allowed to recover from surgery for 1 week prior to the injection of test compound. Data recording on temperature loggers was initiated 48 h prior to injection for calculation of baseline values. Test compound injections (i.v.) were made between 7:00 AM and 9:00 AM by experienced personnel. The data loggers measured the core body temperature of animals at a time interval of 1 min for up to 6 h post-injection. In selected animals, following temperature monitoring, CSF was collected from a direct puncture to the cisterna magna. Blood samples were taken from the tail vein following CSF collection and samples were centrifuged (15 min at 15,000 rpm; room temperature). Samples were stored at −80 °C until analysis. Following blood collection, rats were thoroughly perfused with 10 mL of heparinized (100 U/mL) saline at a rate of 1 mL/min via the left common carotid artery to facilitate specific perfusion of the brain. Brains were then removed and homogenized in an ice-cold homogenization buffer containing 50 mM Tris-HCl pH 8, 150 mM NaCl, and a protease inhibitor cocktail (Sigma-Aldrich, Oakville, ON, Canada) using a Dounce homogenizer (10–12 strokes at 4 °C). Brain homogenates were depleted of vessels using a sequential filtration through 100 and 20 μm nylon Nitex mesh filters (pluriSelect, Leipzig, Germany). Successful vascular depletion of parenchymal fractions was confirmed using the enrichment of a parenchymal marker (Slc1a3) with the concomitant absence of a specific vascular marker (Slc2a1) as previously observed [4]. The concentration of injected antibodies was determined in the vessel-depleted parenchymal fraction using SRM as described below.

Cynomolgus monkeys were surgically implanted with telemetry transmitters (M11 and L11, DSI, St-Paul, MN, USA) at least 12 days prior to dosing. Briefly, the telemetry transmitter was placed between the internal abdominal oblique muscle and the aponeurosis of the transverses abdominis. Test compounds were administered three times (approx. 1 h apart) by IV bolus injection (over 1 to 2 min) in the saphenous vein using a temporary catheter. After the administration, the temporary catheter was flushed with 0.3 mL of saline. Body temperature was recorded continuously from at least 24 h prior to dosing and up to 24 h post-last dose. Blood samples were collected for up to 7 days post-injection and analyzed using SRM as described below.

### 2.6. NanoLC-SRM Mass Spectrometry Analyses

The nanoflow ultrahigh performance liquid chromatography-coupled selected-reaction monitoring (nanoLC-SRM) mass spectrometry method was used to quantify absolute or relative levels of proteins in the serum, cerebrospinal fluid and vessel-depleted brain parenchyma fractions. All protein extracts were reduced, alkylated and trypsin-digested using a previously described protocol [26]. Mass spectrometry analyses were carried out on a NanoAcquity UPLC (Waters, Milford, MA, USA) containing a C18 PepMap™ 100 trap (ThermoFisher, Waltham, MA, USA) followed by a nanoLC BEH130C18 column (Waters, Milford, MA, USA) coupled with ESI-LTQ-XL-ETD or ESI-TSQ-Quantiva mass spectrometers (ThermoFisher Waltham, MA, USA). Peptide signatures of various antibodies were identified by analyzing the respective samples with tandem mass spectrometry (nanoLC-MS/MS) using data-dependent acquisition on ESI-LTQ-XL-ETD44. For the absolute quantification of antibodies, at least 9 standards consisting of calibration and QC standards between 0.05 and 16 fmol range were created by spiking antibodies in their respective control matrices. Each sample was analyzed using nanoLC-SRM and the data was extracted from raw files and analyzed using Skyline 64-bit 20.2.0.286 software (MacCoss Lab Software, University of Washington, Seattle, WA, USA) available as open-source software from https://skyline.ms (last accessed on 19 July 2026).

### 2.7. In Situ Brain Perfusion (ISBP) and Ex Vivo Brain Optical Imaging

A20.1Fc was labeled with the NIR dye CF790-NHSester (ThermoFisher, Waltham, MA, USA). Briefly, A20.1Fc was diluted in carbonate–bicarbonate buffer pH 9.3, then 6:1 molar excess of 10 mM of CF790-NHSester in DMSO was added and incubated for 2 h at room temperature (RT) and then overnight at 4 °C. At the end of the reaction, free, unreacted dye was removed using Zeba Spin Desalting Columns with a molecular weight cutoff of 7 kDa. The final concentration and the degree of labeling (DOL) were determined using the manufacturer’s instructions. ISBP was performed as described previously [27]. Briefly, 4 h after administration of 5 mg/kg of PD149163 (MilliporeSigma, Burlington, VT, USA) BALB/c mice were anesthetized by intraperitoneal injection of xylazine/ketamine (8/140 mg/kg). The right common carotid artery was ligated close to the heart and the right external carotid artery at the bifurcation level to ensure that the perfusate travels directly to the brain through the internal carotid artery. A polyethylene tube filled with heparin (25 IU/mL) was inserted in the right common carotid artery. The perfusion was initiated immediately after cutting off the heart at a flow rate of 2.5 mL/min. A20.1Fc labeled with CF790 was prepared in the following buffer: 128 mmol/L NaCl, 24 mmol/L NaHCO_3_, 4.2 mmol/L KCl, 2.4 mmol/L NaH_2_PO_4_, 1.5 mmol/L CaCl_2_, 0.9 mmol/L MgCl_2_, and 9 mmol/L d-glucose. The buffer was heated to 37 °C after adjusting its pH to 7.4 using a gas mixture of 95% O_2_ and 5% CO_2_. The antibody was perfused for 2 min, followed by 3 min of washout with the same buffer alone.

After performing the ISBP procedure, brains were extracted and scanned ex vivo using an eXplore Optix MX3 pre-clinical imager (Advanced Research Technologies Inc., Montreal, QC, Canada) to measure the fluorescence intensity. The imaging protocol was performed as described previously [27]. The eXplore Optix pre-clinical imager Optiview version 2.02 software (Advanced Research Technologies Inc., Montreal, QC, Canada) was used to reconstruct the images as fluorescence intensity maps. Immediately after the end of imaging, right hemispheres were frozen on dry ice and kept at −80 °C for future homogenization to calculate various brain uptake parameters.

Right hemispheres perfused with A20.1Fc-CF970 were homogenized in lysis buffer (150 mM NaCl, 50 mM Tris and 1% sodium deoxycholate, pH 8) containing a protease inhibitor cocktail (Sigma Aldrich, St. Louis, MO, USA). Black 96-well plates were loaded with equal volumes of perfusates and brain homogenates samples. The sample fluorescence was measured using a BioTek™ FLx800™ Microplate Fluorescence Reader (BioTek, Winooski, VT, USA). Afterward, the distribution volume (VD, μL/g) of the perfused molecule was calculated by dividing the fluorescence concentration in the homogenate of the right hemisphere (Xbrain, fluo/g) by the fluorescence concentration in the perfusate (Cperf, fluo/μL).

### 2.8. PK Analysis in Serum of Non-Human Primates

Plasma concentration–time data for A20.1Fc-neurotensin and FC5Fc-neurotensin were analyzed using naive pooled data and a two-compartment model with IV bolus input, first-order elimination, and macro-rate constants to estimate the following pharmacokinetic parameters: volume of distribution of the central compartment (V1) and the peripheral compartment (V2), clearance (CL), inter-compartmental clearance (CLD), distribution half-life (t1/2α), elimination half-life (t1/2β), and AUC from zero to infinity (AUC0-∞) and maximum predicted concentration (Cmax). Overall, the goodness of fit was based upon the predicted estimate and percent coefficient of variation (% CV) for primary and secondary parameters, as well as the inspection of residual plots between observed and predicted concentration–time data.

### 2.9. Statistical Analysis

The results are expressed as the mean ± SEM or SD as indicated. Where applicable, a paired *t*-test was used. One-way ANOVA followed by Newman–Keuls’ post-test was used to compare multiple groups. A *p*-value of less than 0.05 was considered statistically significant.

## 3. Results

### 3.1. Characterization of A20.1Fc and FC5Fc Fused with Neurotensin

We expressed A20.1Fc and FC5Fc constructs with a C-terminus fusion of neurotensin in stable CHO cell lines. SEC profile (Figure 1A) and SDS-PAGE analysis (Figure 1B) indicate that the obtained proteins were highly pure. Quality attribute characterization also indicated that the constructs were obtained in suitable concentrations for in vivo work with low levels of endotoxin (Table 1).

We then tested whether FC5Fc-neurotensin and A20.1Fc-neurotensin were able to activate NTS1 in a cell-based assay (Figure 2A). Although a slight rightward shift in the concentration–response curves was observed for both fusion proteins compared with the 13 amino-acid neuropeptide alone, this difference was not statistically significant. Interestingly, when A20.1Fc-Neurotensin and FC5Fc-Neurotensin were expressed in transiently transfected CHO-3E7, a C-terminal truncation of the neurotensin sequence was revealed, resulting in the loss of the terminal Ile-Leu residues (Figure 2B). Given that the receptor-interacting residues of neurotensin are at its C-terminus it is not surprising that the truncated constructs exhibited impaired NTS1 activation in vitro (Figure 2A). Overall, our data support the feasibility of producing antibodies–neurotensin fusion proteins while highlighting the importance of rigorous quality control of the material as C-terminus clipping of neurotensin can occur and compromise the functional activity of the fusion proteins.

### 3.2. Changes in Core Temperature Following Administration of Neurotensin Alone or Conjugated with A20.1Fc and FC5Fc

NT, a 13-amino-acid neuropeptide, and its receptors are widely distributed in the CNS [21]. It has been shown that central administration of NT leads to a sustained decrease in the core body temperature through NTS1-mediated mechanisms [22]. However, due to its structure, NT is unable to cross the BBB, and we confirmed that administration of neurotensin alone (1.79 µmol/kg or 3 mg/kg) does not induce any changes in the core temperature in mice (Figure 3A). Similarly, when NT was fused with A20.1Fc, no effect was observed following an intravenous injection of 241 nmol/kg (equivalent to 20.06 mg/kg) (Figure 3B), which is consistent with its inability to cross the BBB. Next, we determined whether FC5 is capable of delivering a pharmacologically active payload into the brain of mice. Systemic administration of FC5Fc-Neurotensin (241 nmol/kg or 19.99 mg/kg) induced a consistent and prolonged decrease in the core temperature in mice that lasted for up to 5 h post-injection (Figure 3C). Although it is assumed that the transport across the BBB initiates immediately following the intravenous administration, the drop in core temperature following FC5Fc-Neurotensin administration was not apparent until approximately 20 min post-injection. One possible explanation is stress-induced hyperthermia resulting from the restraint procedure required to administer the test compounds, which may transiently mask the hypothermic effect during the early post-injection period. Consistent with this interpretation, a transient spike in core temperature was observed following NT and A20.1Fc-Neurotentin administration in mice, highlighting the need for extensive habituation protocols and personnel training to minimize this issue.

We then proceeded with a dose-dependent analysis to characterize the hypothermic effects of the antibodies fused to neurotensin. To further demonstrate the translation of this model we selected to perform these studies in rats. Animals received increasing doses of neurotensin alone (1.19 µmol/kg and 2.98 µmol/kg, 2 mg/kg and 5 mg/kg, respectively), A20.1Fc-neurotensin (120 nmol/kg and 241 nmol/kg) and FC5Fc-neurotensin (120 nmol/kg and 241 nmol/kg) (Figure 4A–C). Similarly to what was observed in mice, systemic administration of FC5Fc-neurotensin, but not neurotensin alone or A20.1Fc-neurotensin, induced a time and dose-dependent drop in core temperature.

To quantitatively characterize the hypothermic response, we derived several parameters from the temperature–time profiles. First, we defined the baseline core temperature (Tb) as the average temperature measured in undisturbed animals 24 h prior to test compound administration. Measurements were collected at the same time period as dosing to minimize variability due to the fluctuations in core body temperature present during the circadian cycle. The first parameter we evaluated was the maximum effect, defined as the maximum decrease in core temperature relative to baseline, calculated as difference between Tb and the minimum temperature recorded after test compound administration (Figure 4D). We then analyzed the duration of the hypothermic response (Figure 4D). The average temperature (Tb) and its standard deviation (SD) were used to calculate the duration of response. Hypothermia duration was defined as the time during which the core temperature was below Tb–2SD during the interval from dosing to the last measurement. Finally, the area under the curve (AUC) was calculated using a trapezoidal rule from 0 to 360 min observation period (Figure 4D). Analysis of the maximum effect, area under the curve and duration of response demonstrated statistically significant hypothermic effects following systemic administration of FC5Fc-neurotensin. Collectively, these findings further demonstrate that FC5Fc is capable to transport a pharmacological active payload across the BBB and into the brain after systemic administration.

We confirmed the BBB permeability of our carrier by NanoLC-SRM mass spectrometry in CSF and vessel-depleted brain parenchyma fraction from tissues harvested 6 h post-injection of A20.1Fc-neurotensin or FC5Fc-neurotensin at a dose of 241 nmol/kg (Figure 4E). Whereas serum levels of both constructs were similar (2757 ± 164 nM vs. 2844 ± 195 nM, A20.1Fc-neurotensin and FC5Fc-neurotensin, respectively), both the CSF (0.33 ± 0.06 nM vs. 1.66 ± 0.22 nM) and brain (1.27 ± 0.37 nM vs. 11.13 ± 2.01 nM) levels of FC5Fc-neurotensin were significantly increased when compared with A20.1Fc-neurotensin, further highlighting the capacity of FC5 construct to be transported across the BBB.

### 3.3. Effect of Hypothermia on BBB Integrity

Although the mild hypothermia induced in our model is generally considered to be protective of BBB function, especially after insults like ischemic damage [28], we still questioned whether the sudden changes in core temperature could alter the BBB integrity. To address this possibility, the mice were injected with a high dose (5 mg/kg) of PD149163, a BBB-permeable NTS1 agonist (Figure 5A). As expected, PD149163 induced a significant drop in the core body temperature (minimum temperature 28.14 ± 0.41) that lasted for up to 9 h (Figure 5A). BBB integrity was determined by in situ brain perfusion 4 h post-PD149163 administration, when the core temperature of mice had started to return back to baseline levels. For these experiments, A20.1Fc was labeled with CF790. The CF790 dye was added at predetermined molar ratios to achieve a target dye-to-protein (D/P) ratio of approximately three fluorophores per antibody molecule in order to minimize over-labeling, which could potentially affect protein structure and function. Our results indicate that there is no difference in A20.1Fc-CF970 permeability between PD149163-treated mice and saline-treated controls, indicating that the hypothermia induced by PD149163 did not measurably affect BBB integrity (Figure 5B,D). The functionality of the in situ brain perfusion technique was confirmed by comparing A20.1Fc brain accumulation under intact and osmotically disrupted BBB conditions. Osmotic disruption with 22% mannitol provides an approximation of the maximum achievable accumulation of the test antibody. As shown in Figure 5C,D, perfusion with A20.1Fc-CF970 in mannitol resulted in a marked increase in the fluorescent signal in the right hemispheres compared to the fluorescence of A20.1-Fc-CF970 with perfusion in saline. Taken together, these results suggest that the acute hypothermia induced in our model does not measurably affect the BBB integrity.

### 3.4. Neurotensin-Induced Hypothermia in NHP

NHPs are important for translational research given their close genetic, physiological, and behavioral similarity to humans versus rodents. We next questioned whether the neurotensin-induced hypothermia model could be extended to NHP as a functional pharmacodynamic readout for the pre-clinical development of BBB carriers. Due to the limited information regarding the safety and pharmacological effects of administering antibody–neurotensin fusion protein in NHPs, we adopted a conservative dosing regimen. Animals received three intravenous doses of the test compounds (120 nmol/kg per dose), administered 60 min apart (Figure 6A). Blood samples were collected for up to 7 days post-injection for pharmacokinetic analysis. Intravenous administration of FC5Fc-neurotensin induced a transient drop in the core body temperature following the second and third injection (Figure 6B). This result possibly suggests that a minimum brain exposure is required to achieve a quantifiable drop in the pharmacodynamic behavior in NHP. Nonetheless, statistical analysis indicates that the maximum effect, area under the curve and duration of response of FC5Fc-neurotensin differ significantly from the response to A20.1Fc-neurotensin (Figure 6D). Interestingly, a similar dosing regimen in rats produced an initial transient decrease in the core temperature, followed by a prolonged hypothermic response (Figure 6E). Although a variability in core temperature is observed in NHPs injected with A20.1Fc-neurotensin, the values did not decrease below the pre-injection or baseline levels (Figure 6C). We believe that this oscillation is likely caused by stress-induced hyperthermia due to the repeated cycles of animal restraining during blood collection and test compound administration. In fact, similar transient increases in the core temperature were observed in other time-course curves throughout this study, highlighting the potential influence of handling related stress and the importance of experimental design and operator experience when implementing this model. Finally, the serum PK profiles of A20.1Fc-neurotensin and FC5Fc-neurotensin are shown in Figure 6G. Both fusion proteins exhibited a biphasic decrease in serum concentrations which was fitted to a two-compartment PK model. The serum PK parameters are summarized in Table 2. Despite the fact that statistically significant differences in pharmacokinetic parameters could not be determined due to the very small sample of animals in each group, there is a clear trend for FC5Fc-Neurotensin to have a lower exposure (~20%) relative to A20.1Fc-Neurotensin. This is reflected by AUC0−∞ values of 65 nmol×h/mL vs. 85 nmol×h/mL for FC5Fc-Neurotensin and A20.1Fc-Neurotensin, respectively. These differences support that the drop in the core body temperature from baseline (Figure 6B,C) observed with FC5Fc-neurotensin is not the result of the difference in systemic exposure but to an enhanced BBB-crossing from the presence of FC5. Of interest, these PK values were within the range previously reported in the literature for BBB-crossing antibodies targeting the transferrin receptor in non-human primates [29].

## 4. Discussion

The present study provides an advanced characterization of the brain permeability and CNS delivery potential of FC5Fc, a BBB carrier, using the neurotensin-induced hypothermia model. In addition to evaluating FC5 in rats and mice, the two species most commonly used in pre-clinical research, we also demonstrate that FC5Fc-neurotensin can elicit a pharmacological response following systemic administration in non-human primates. These findings extend the characterization of FC5Fc across species and provide further support for its potential application as a platform for CNS drug delivery.

The development of CNS therapeutics is severely hampered by poor tissue distribution due to the BBB. Therefore, overcoming or exploiting naturally occurring transport pathways present in this barrier is a prerequisite to achieve sufficient exposure of therapeutic agents to their CNS targets and elicit a pharmacological response. Whereas the BBB acts as an important defense system, limiting the entry of potentially harmful circulating substances into the brain, it simultaneously restricts the entry of most therapeutic agents [1]. Under physiological conditions, essential macromolecules and nutrients are transported across the BBB through both receptor-mediated transcytosis-dependent and -independent mechanisms [30]. These endogenous transport pathways have consequently been exploited to facilitate the delivery of CNS therapeutics by targeting receptors expressed at the BBB and engaging RMT pathways [4,5,6,7,8]. We have previously shown that single-domain antibodies, which are naturally occurring single-variable heavy-chain fragments of heavy-chain IgGs found in camelid species, can possess BBB-transmigrating properties. Among them, FC5 has been shown to transmigrate across the brain endothelial cell monolayer via a saturable, directional, energy- and microtubule-dependent pathway, triggered by antibody internalization via clathrin-coated vesicles [6,31].

The use of Chinese hamster ovary (CHO) cells coupled with selectable expression systems has revolutionized the manufacture of novel protein-based therapeutics by enabling the rapid and scalable production of large quantities of recombinant proteins [32]. In addition to their well-documented safety profile and suitability for large scale-up in bioreactors, CHO cells support complex post-translational processing, which can be easily monitored during recombinant protein production [33]. Amongst the potential post-translational modifications commonly encountered in biologics, variation in C-terminal sequence resulting from proteolytic clipping by carboxypeptidases is frequently observed in therapeutic antibodies [34]. This is of particular importance for neurotensin constructs because structure-activity studies have shown that the C-terminal hexapeptide (8–13) contains the structural requirements for high-affinity binding and full agonist activity at the NTS1 receptor [35]. We, therefore, confirmed the functional activity of the antibody–neurotensin fusion proteins using a cell-based NTS1 activation assay. Both FC5Fc-neurotensin and A20.1-neurotensin displayed similar concentration–response profiles and EC50, with only a modest rightward shift relative to neurotensin alone (Figure 2A). Interestingly, C-terminal clipping was noted in one batch of fusion proteins and confirmed by intact mass spectrometry (Figure 2B). As expected, these constructs showed blunted activation of the NTS1 and were thus discarded (Figure 2A). Reasons for intact protein production in some cases and the C-terminus clipping in other batches are not clear. CHO cells express different carboxypeptidases that can contribute to C-terminal processing of recombinant proteins. For instance, carboxypeptidase D has been implicated in the C-terminal lysine clipping of monoclonal antibodies produced in these cells [36]. Meanwhile, carboxypeptidase A was shown to be the enzyme responsible for the rapid degradation of neurotensin to the inactive 11 amino acid fragment (NT_1–11_), although a potential contribution of carboxypeptidase D cannot be excluded [37]. Additionally, metallopeptidases have also been shown to inactivate neurotensin. Endopeptidase 24.15 hydrolyzes neurotensin at the Arg^8^-Arg^9^ bond, whereas endopeptidase 24.16 cleaves it at the Pro^10^-Tyr^11^ bond. Endopeptidase 24.11, also known as neprilysin or neutral endopeptidase, cleaves neurotensin at both the Pro^10^-Tyr^11^ and Tyr^11^-Ile^12^ bonds [38]. According to our intact mass spec analysis, the truncated constructs displayed a significant percentage of antibodies missing the Ile^12^-Leu^13^ or Leu^13^ (Figure 2B) possibly suggesting a role of these endopeptidases in the generation of constructs with impaired NTS1 activation capacity.

The translational path for RMT-based strategies ultimately requires in vivo animal testing to establish functional efficacy [39]. This is particularly important because biodistribution and pharmacological activity in vivo are governed by numerous intertwined factors including blood flow dynamics, tissue and cellular barriers, hormonal regulation, filtration and metabolic clearance, among others. Pharmacodynamic models can therefore provide valuable functional evidence that carriers can achieve transcytosis across the BBB and deliver a payload into the brain at levels sufficient to elicit a measurable pharmacologic response. Disease-relevant models, such as those examining β-amyloid clearance, have been used to demonstrate the uptake of carriers at pharmacologically relevant doses [40]. However, such models can be complex, time-consuming and dependent on the specific therapeutic mechanism being investigated. An alternative approach is to validate the ability of an RMT target to mediate CNS delivery using a pharmacodynamic readout that is independent of a disease outcome. The neurotensin-based hypothermia represents an attractive assay platform for rapid functional validation of brain parenchymal uptake of multiple potential BBB carriers targeting different receptors [4,14,15,16,17,18,19,23]. Neurotensin is an endogenous neuropeptide produced by neurons and glial cells in the brain where it functions as a neurotransmitter and neuromodulator involved in a wide range of physiological processes, including body temperature, blood pressure, and nociception [21]. Neurotensin has negligible BBB permeability and when administered peripherally no CNS-mediated effects are observed as shown in Figure 3A and Figure 4A and in previous studies [4,14,15,16,17,18,19,22]. In contrast, when NT is fused to the C-terminus of the BBB-crossing FC5Fc, systemic administration produced a rapid reduction in the core temperature in both rats and mice (Figure 3 and Figure 4). Importantly, as expected, no changes in the core temperature were observed when NT was fused to A20.1Fc, a control non-BBB-crossing antibody (Figure 3 and Figure 4). Beyond the presence or absence of a hypothermic response, additional parameters can be derived from the temperature–time profiles, such as maximum effect, duration of response, and area under the curve. In our study, all three parameters differentiated FC5Fc-neurotensin from the negative controls, including neurotensin alone or fused with A20.1Fc, and further demonstrated a dose-dependent response to FC5Fc-neurotensin (Figure 4D). This quantitative approach provides a more comprehensive assessment of the pharmacodynamic activity associated with CNS delivery and may be particular useful for comparing and ranking the relative performance of different BBB carriers.

One major limitation of the NT-induced hypothermia model is that the response is mediated primarily by hypothalamic neurons and therefore may not necessarily reflect the ability of the BBB carrier to deliver its cargo to other regions of the brain [41]. Previously, we have demonstrated that positive staining for FC5 is observed in sections of the thalamus, parietal cortex, caudate putamen and hippocampus following systemic administration [42]. Additional evidence that FC5 is transported across multiple areas of the brain is provided when FC5 was fused with an amyloid-β oligomer-binding peptide and administered to an animal model of the familial form of Alzheimer’s disease. In this study there was a significant reduction in the amyloid-β loading in the prefrontal cortex, cingulate cortex, entorhinal cortex, associative cortex, retrosplenial cortex, nucleus accumbens, and hippocampus areas of the brain [43]. Taken together, these results support the notion that FC5 is able to penetrate multiple regions of the brain and is an optimal candidate for clinical use in various disease applications. To further confirm the capacity of FC5Fc to shuttle macromolecular cargoes across the BBB we performed MRM mass spectrometry analysis in capillary-depleted whole brain homogenates and CSF samples 6 h post-injection (Figure 4E). At this time point, a significant accumulation of FC5Fc-neurotensin, when compared to A20.1Fc-neurotensin, was observed in both brain parenchyma (9-fold) and CSF (5-fold). The concentrations of FC5Fc-neurotensin detected in the brain were comparable to those reported for TfR-targeting constructs that induced a hypothermic response when fused with neurotensin [23]. Interestingly, consistent with previous reports, the core body temperature began to recover within 2 to 3 h despite sustained elevation of FC5Fc-neurotensin brain levels at the 6-h timepoint. This dissociation between brain exposure and pharmacodynamic response suggests that the duration of hypothermia is not determined solely by the concentration of the fusion protein in the CNS. Receptor desensitization and/or proteolytic cleavage of the neurotensin payload may contribute to this effect [44,45]. We also questioned whether the sudden drop in core temperature could disrupt the integrity of the BBB and therefore confound the interpretation of the model. Using in situ brain perfusion, we assessed the brain permeability of A20.1Fc following a prolonged hypothermic challenge induced by PD149163, a small molecule NTS1 agonist that is BBB-permeable. Despite the marked decrease in body temperature, A20.1Fc remained largely excluded from the CNS (Figure 5). In fact, increased fluorescence of A20.1Fc was observed only when the BBB was osmotically disrupted. These findings indicate that the hypothermic response itself does not measurably disrupt BBB integrity, further supporting the use of the neurotensin model to differentiate BBB-crossing from non-crossing molecules (Figure 5C,D).

Cross-species differences in the expression of RMT receptors at the BBB and peripheral tissues are an important consideration in translational development of BBB carriers. Although the expression profiles of many transporters at the primate BBB are generally considered to be similar to those in humans, several RMT receptors are expressed at substantially higher levels at the mouse BBB than at the human BBB [46]. This issue highlights the importance of evaluating the functional properties of the BBB carrier across multiple species rather than relying on rodent models only [46,47]. To this end, we tested the functional CNS delivery of FC5Fc-neurotensin in NHPs and characterized the corresponding serum pharmacokinetic profile (Figure 6). Systemic administration of FC5Fc-neurotensin produced a measurable decrease in core body temperature, whereas no comparable response was observed following the injection of A20.1Fc-neurotensin, suggesting that the carrier successfully transports an active payload into a larger and much more complex central nervous system (Figure 6B). Of interest, the magnitude of the response was within the range reported for the brain-penetrating neurotensin agonist NT69L administered to female rhesus monkeys [48]. It should be noted that the hypothermic response in NHP was very mild and only measurable after the second and third injection of FC5Fc-neurotensin at 120 nmol/kg, with a maximum decrease of approximately 0.6 °C from the baseline after the second injection and 0.85 °C after the third. In comparison, rats administered an analogous regimen (three injections of 120 nmol/kg, 60 min apart) exhibited a substantially more pronounced and long-lasting effect (Figure 6E). Differences in the magnitude and duration of the response between rodents and NHPs are not unexpected given fundamental interspecies differences in thermoregulatory physiology, body size and thermal inertia resulting in large animals generally exhibiting greater thermal stability and slower changes in core temperature than smaller mammals [49]. In fact, key determinants in thermoregulation, including surface area to mass ratio, metabolic rate normalized to body mass, relative abundance of brown adipose tissue, and whole-body thermal conductance differ significantly across species [50]. Small mammals such as a rat or a mouse with a relatively large surface area to volume ratio and high thermal conductance rely more on metabolic thermogenesis in brown adipose tissue (BAT) to regulate the core temperature [48]. In contrast, as body size increases, peripheral vasomotor control mechanisms become increasingly important for thermoregulation, and animals with significant muscle mass have also the capacity to combat an acute, strong cold challenge by generating more heat from shivering [51,52]. These physiological differences likely contribute to the reduced magnitude of the NT-induced hypothermic response observed in NHPs and should be considered when using temperature changes as a pharmacodynamic endpoint for cross-species comparison. It should also be emphasized that within the same species and strain, hormone regulation plays an important role in thermoregulation. It has been shown that estrogen-sensitive neurons in the medial preotic area of the hypothalamus drive the thermoregulatory and metabolic responses to energy deficiency directly affecting the minimum core temperature and variation in the duration of hypothermia [53]. In order to reduce this source of variability, in our study we opted to test FC5Fc-neurotensin and A20.1Fc-neurotensin in males only; however, it could be particularly interesting to include female cohorts in similar future studies as well.

Taken together, our findings support neurotensin-induced hypothermia as a convenient functional assay for evaluating BBB carrier activity in vivo. While the assay does not directly quantify the extent or mechanism of transcytosis, the generation of a pharmacological response following peripheral administration provides evidence that the carrier can deliver a functionally active payload to the CNS. In future studies, quantitative analysis of maximum effect, duration of response, and AUC could therefore be used as complementary pharmacodynamic measures to rank and prioritize BBB carriers according to their functional brain-delivery capacity.

## 5. Conclusions

In conclusion, RMT-based approaches have shown significant potential for improving brain drug bioavailability and enhancing the efficacy of a wide array of drugs. In the present study, we provided further support for the development of FC5, a single domain antibody targeting TMEM30A at the BBB, as a promising carrier for CNS drug delivery. Using the neurotensin-induced hypothermia as a functional pharmacodynamic readout, we demonstrated that FC5Fc can transport a pharmacologically active payload across the BBB following systemic administration in mice, rats, and non-human primates.

We also demonstrate that the neurotensin-induced hypothermia model can be considered an efficient screening assay to determine the BBB permeability of candidate carriers through target-engagement of NTS1 expressed by hypothalamic neurons. Main advantages of this method include its user-independent readout, through implantation of telemetry devices, and the fact that it is translatable across multiple species with significant differences in brain size, anatomy, complexity and physiology. Importantly, combining the hypothermic response with quantitative measures of brain exposure and appropriate BBB-integrity controls provides a robust framework for evaluating and prioritizing BBB carriers. This is of particular importance when the ultimate goal of the research is to bridge the translational gap between pre-clinical models and the deployment of this technology into the clinic.

## Figures and Tables

**Figure 1:**
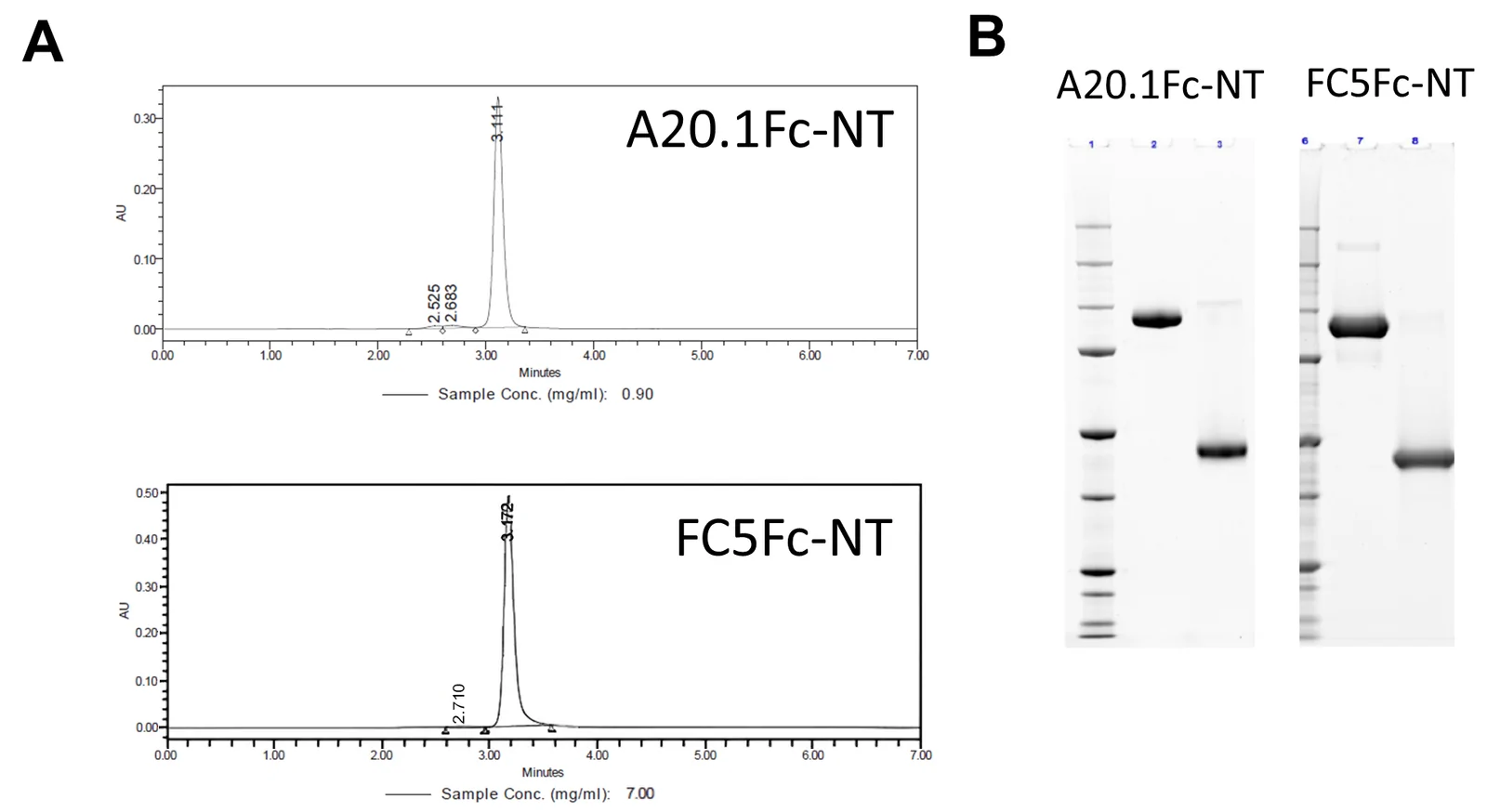
Protein expression profile of neurotensin fusion constructs. (**A**) Size-exclusion chromatography profiles of FC5Fc-Neurotensin and A20.1Fc-Neurotensin. (**B**) An aliquot of each antibody was loaded onto SDS-PAGE and run under non-reduced and reduced conditions.

**Figure 2:**
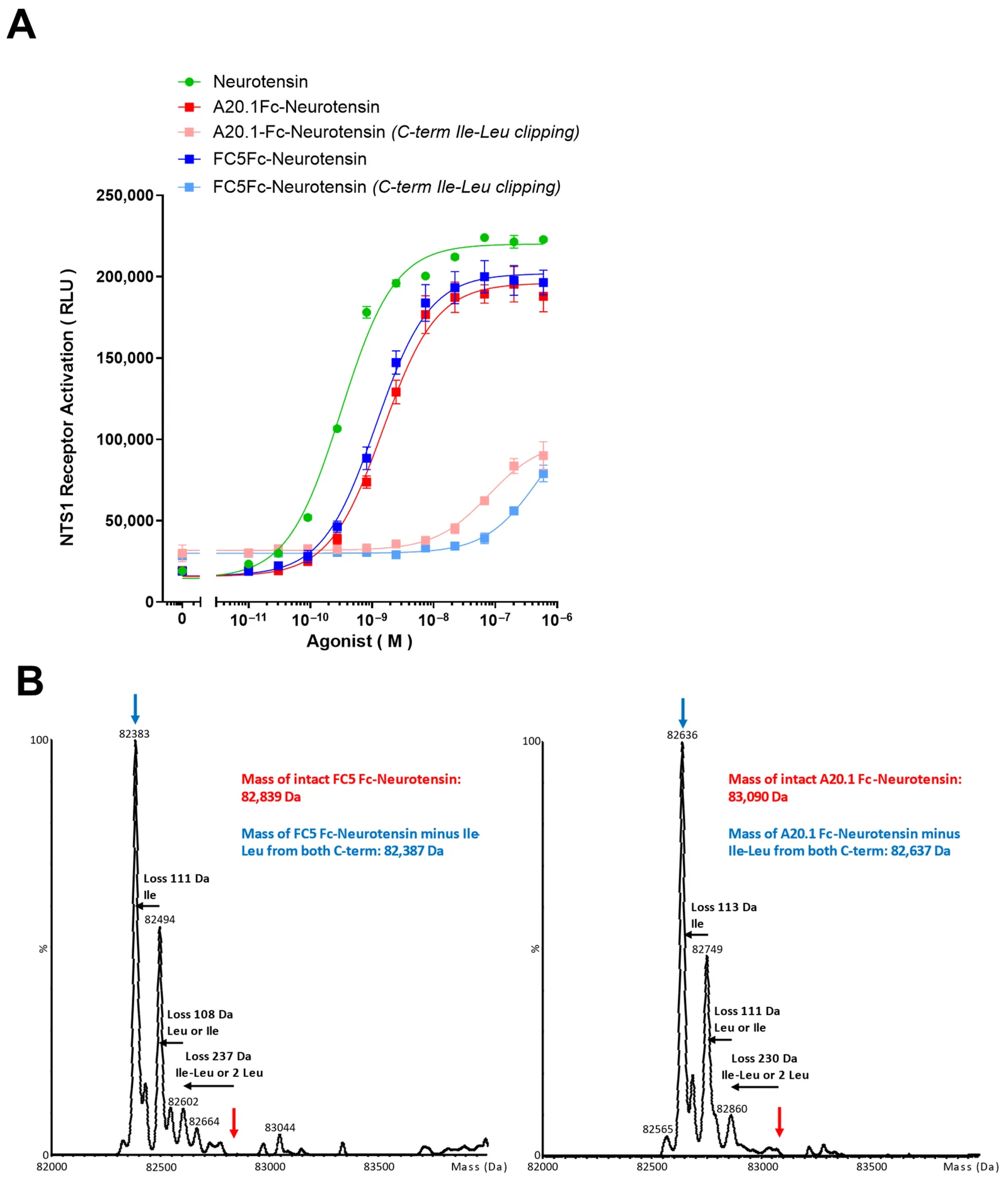
Neurotensin receptor type 1 activation and intact protein LC-ESI-MS analysis of constructs. (**A**) Concentration–response of NTS1 activation induced by neurotensin, FC5Fc-neurotensin, A20.1Fc-neurotensin and constructs that were identified to display significant C-terminus Ile-Leu clipping during protein production with the DiscoveRx PathHunter system in a CHO-K1 cell line. Data is represented as means ± SEM of two wells. (**B**) Molecular weight profile of deglycosylated FC5Fc-neurotensin dimer and A20.1Fc-neurotensin dimer, from intact protein LC-ESI-MS analysis. No signal was observed at the expected mass of the intact protein as indicated by the red arrows but masses were observed matching the protein with loss of Ile-Leu from the C-terminus of one or both chains. Expected masses were calculated assuming formation of all disulfide bonds, deamidation of the N-glycosylation site during deglycosylation by PNGaseF, and pyroQ formation at the N-terminus of A20.1Fc-neurotensin. Loss of Ile-Leu from one chain or loss of Leu from both chains cannot be discriminated by mass alone.

**Figure 3:**
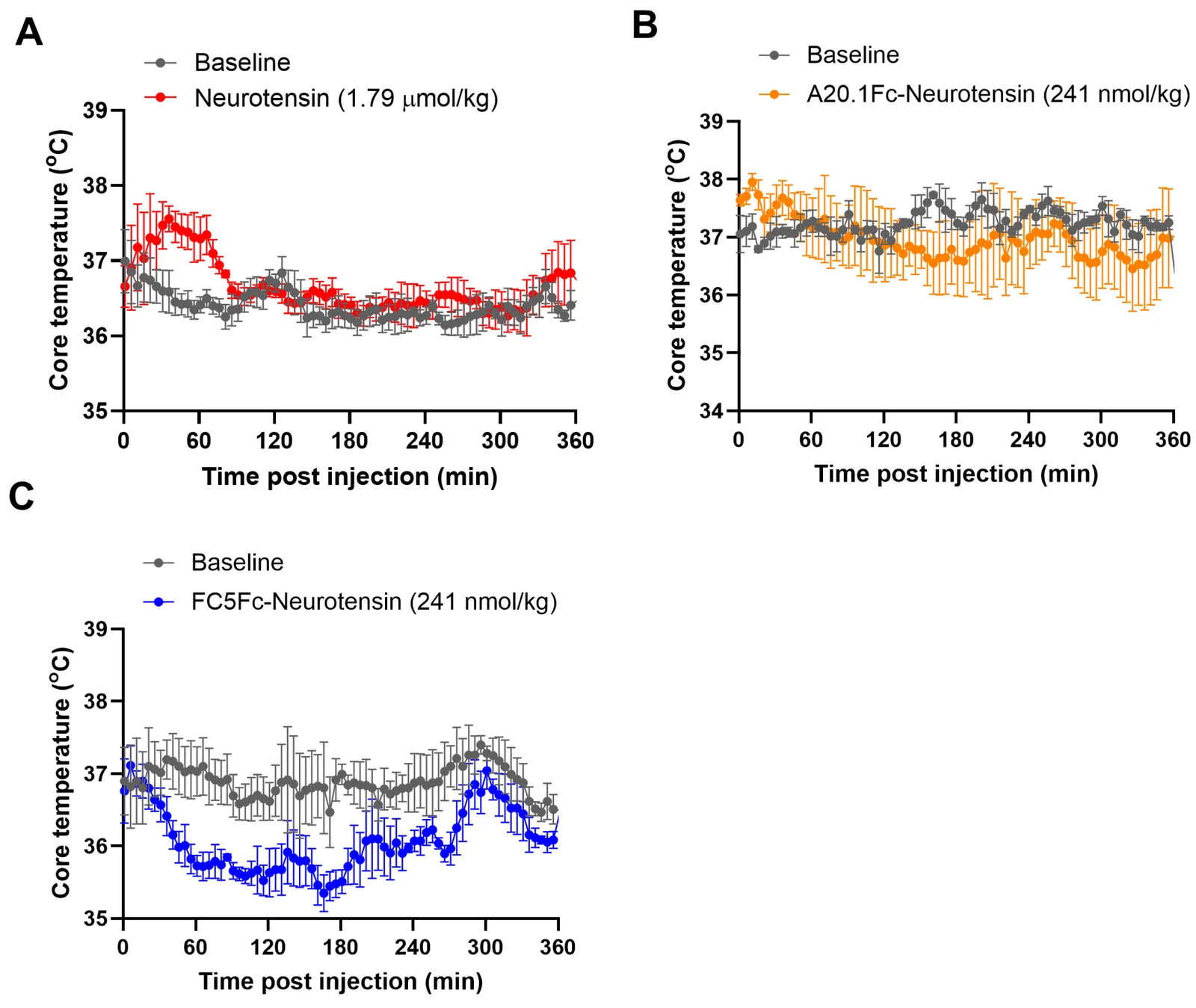
Time-course effect of neurotensin, A20.1Fc-neurotensin and FC5Fc-neurotensin on core temperature of mice. Mice were intravenously injected with (**A**) neurotensin (1.79 µmol/kg, equal to 3 mg/kg, *n* = 4), (**B**) A20.1Fc-neurotensin (241 nmol/kg, equal to 20.06 mg/kg, *n* = 3), or (**C**) FC5Fc-neurotensin (241 nmol/kg, equal to 19.96 mg/kg, *n* = 5). Core body temperature was monitored using telemetry for up to 6 h post-injection of test compounds. Results are shown as mean ± SEM of 3–5 animals.

**Figure 4:**
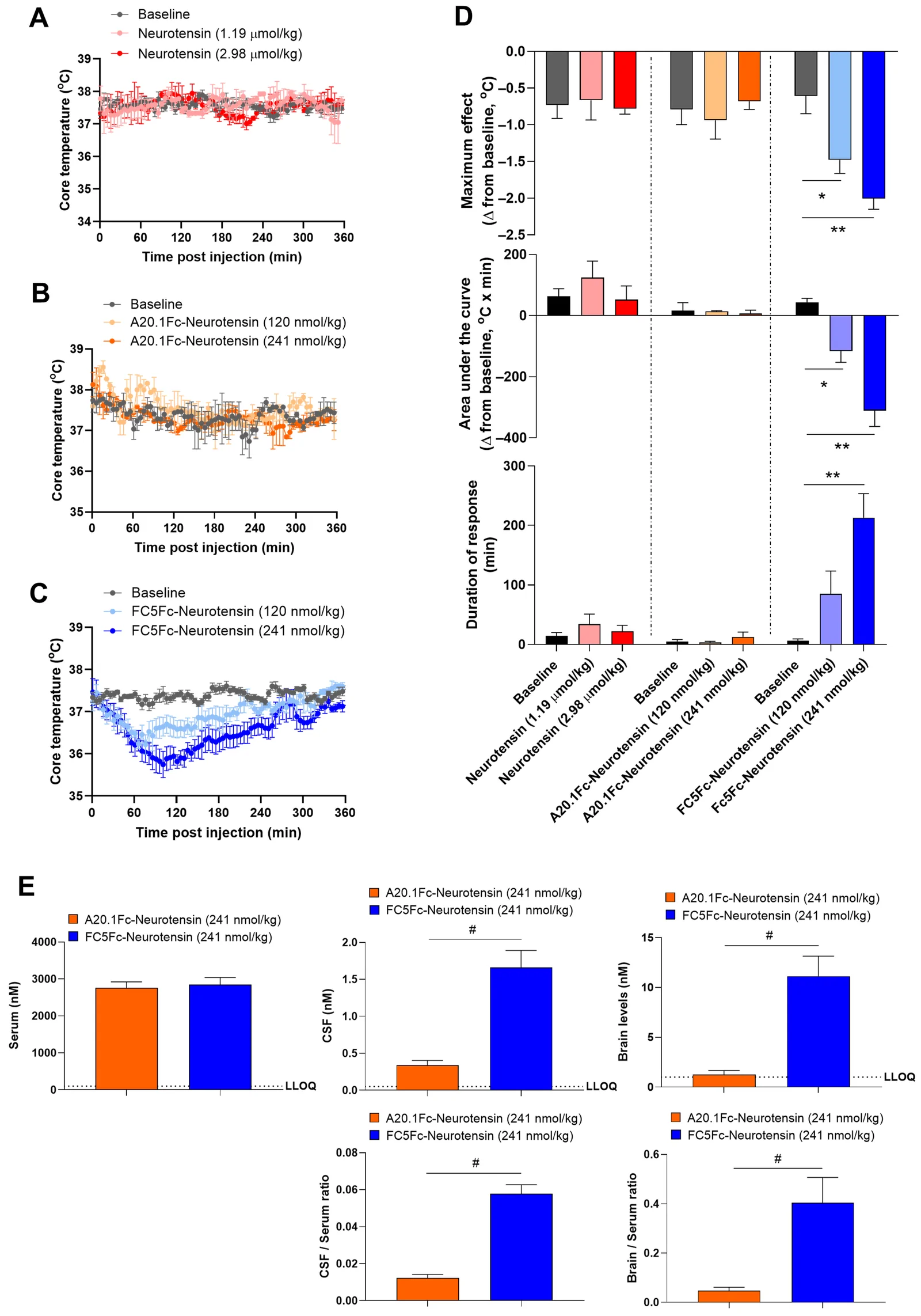
Dose–response analysis and CNS quantification of BBB carriers conjugated to neurotensin in rats. Rats were intravenously injected with (**A**) neurotensin (1.19 µmol/kg, *n* = 3 or 2.98 µmol/kg, *n* = 3), (**B**) A20.1Fc-Neurotensin (120 nmol/kg, *n* = 3 or 241 nmol/kg, *n* = 4), or (**C**) FC5Fc-Neurotensin (120 nmol/kg, *n* = 5 or 241 nmol/kg, *n* = 5). Core body temperature was monitored using telemetry for up to 6 h post-injection of test compounds. (**D**) From the time-course curves, maximum effect, area under the curve and duration of response were extracted. (**E**) At the end of the time course, selected animals injected with 241 nmol/kg of A20.1Fc-Neurotensin (*n* = 3) or FC5Fc-Neurotensin (*n* = 3) were perfused for tissue collection. Serum, CSF and vessel-depleted brain fractions were analyzed using multiplexed nanoLC–SRM-ILIS. Results are shown as mean ± SEM of 3–5 animals. * *p* < 0.05 vs. baseline; ** *p* < 0.001 vs. baseline; # *p* < 0.01 vs. A20.1Fc-Neurotensin.

**Figure 5:**
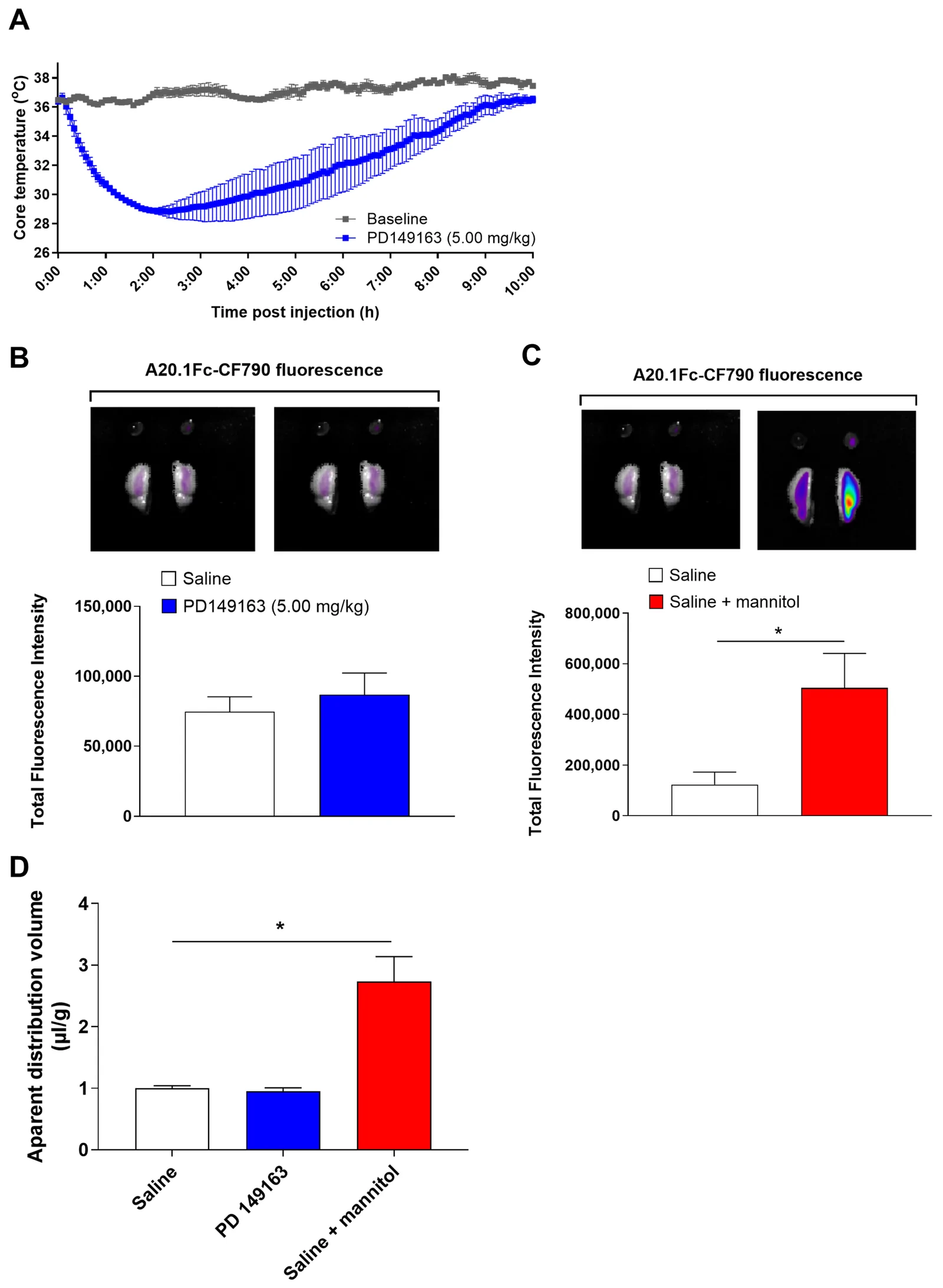
Hypothermia effect on BBB integrity. (**A**) Hypothermic effects of a 5 mg/kg dose of PD149163 (i.v.), a small molecule BBB-permeable NTS1 agonist in mice (*n* = 4). (**B**) In a separate group of animals (*n* = 6), 4 h after PD149163 administration, brain uptake of A20.1Fc labeled with CF790 was measured by in situ brain perfusion. Top panel: Ex vivo optical images of left and right brain hemispheres of mice perfused with A20.1Fc-CF790. Bar graphs represent the quantification of the total fluorescent intensity measured in the homogenates of the right hemisphere. (**C**) Brain uptake of A20.1Fc labeled with CF790 measured 4 h after PD149163 administration in a cohort of animals (*n* = 5) that had the BBB disrupted with a solution of mannitol 22%. Top panel: Ex vivo optical images of left and right brain hemispheres of mice perfused with A20.1Fc-CF790. Bar graphs represent the quantification of the total fluorescent intensity measured in the homogenates of the right hemisphere. (**D**) Apparent distribution volume VD (µL/g) measured in the homogenates of the right hemispheres of mice perfused with A20.1Fc-CF790 in the presence or absence of mannitol 22%. Data was normalized by subtracting background values obtained from mice perfused with equimolar concentration of dye alone. * *p* < 0.05 vs. saline infused mice.

**Figure 6:**
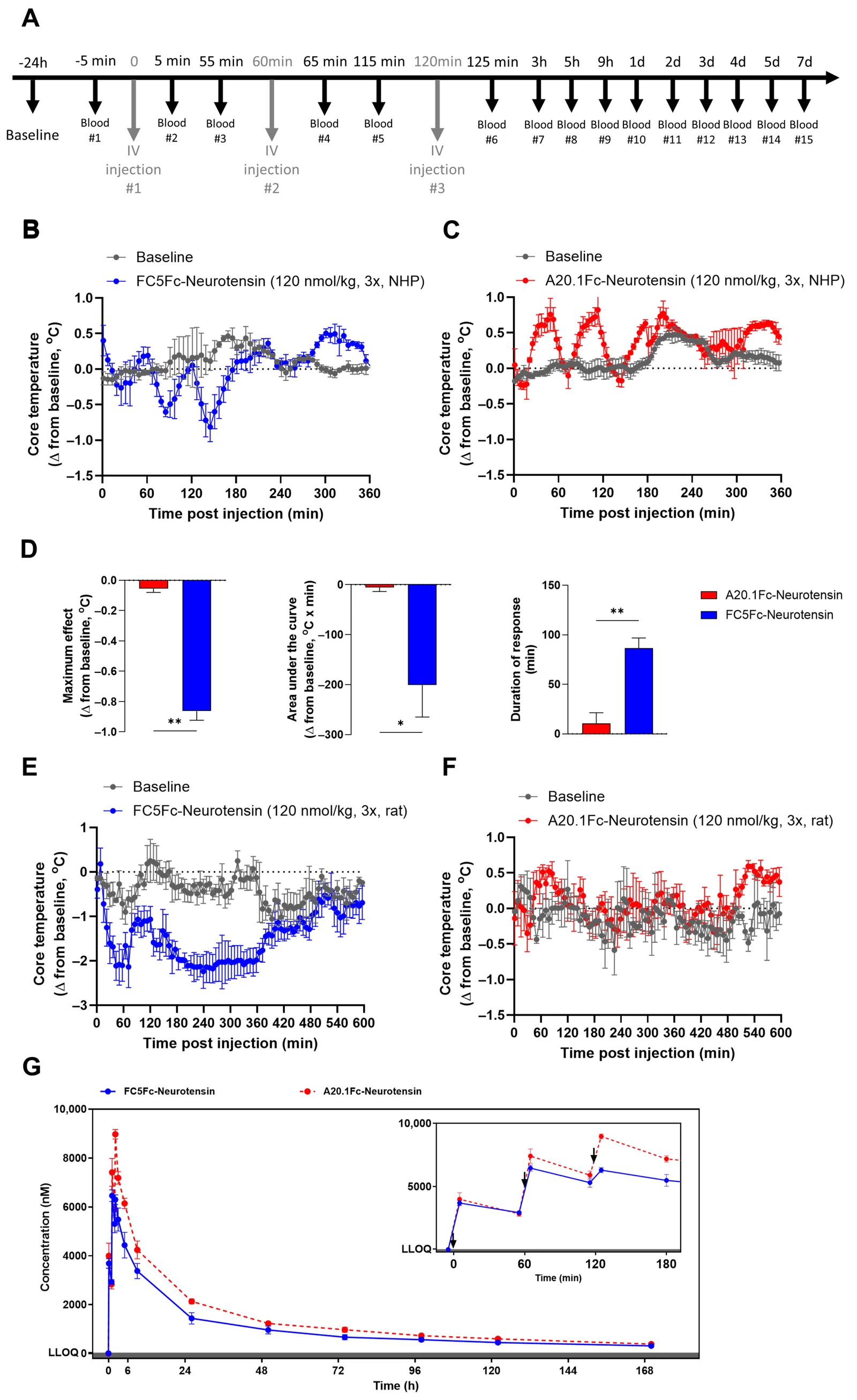
Time-course effect of A20.1Fc-Neurotensin and FC5Fc-Neurotensin on core temperature of NHP and rats. (**A**) Schematic representation of dosing and blood sampling schedule. Cynomolgus monkeys (**B**,**C**) or rats (**E**,**F**) were injected with FC5Fc-Neurotensin (**B**,**E**) or A20.1Fc-Neurotensin (**C**,**F**) with a dose of 120 nmol/kg by intravenous administration three times, 1 h apart. Core body temperature was monitored using telemetry for up to 6 h in NHPs and 10 h in rats post-test compound injection. (**D**) From the NHP time-course curves, maximum effect, area under the curve and duration of response were extracted. Results are shown as mean ± SEM of 3 (NHP studies) or 5 (rat studies) animals. * *p* < 0.05 vs. A20.1Fc-neurotensin; ** *p* < 0.005 vs. A20.1Fc-neurotensin. (**G**) Serum concentration of FC5Fc-Neurotensin or A20.1Fc-Neurotensin injected by intravenous administration three times, 1 h apart. Serum was collected at indicated time points and analyzed using multiplexed nanoLC–SRM-ILIS method. Graph insert contains the detailed serum profile concentration (in nM) and test compound administration (arrows) during the first 180 min of the study.

**Table 1:** Quality attributes of A20.1Fc-Neurotensin and FC5Fc-Neurotensin at harvest and post protein A purification.

Construct	Clarified Harvest		Purified Product			
	Amount (mg)	Volume (mL)	Amount (mg)	Concentration (mg/mL)	% Monomer Content (SEC-UPLC)	Endotoxin (EU/mg)
A20.1Fc-Neurotensin	746.6	395	314.4	5.765	99.5	<0.017
FC5Fc-Neurotensin	1456.8	607	1006.9	6.978	99.4	<0.0072

**Table 2:** Mean PK parameter estimates and %CV from 2-compartment analysis of serum concentration–time profiles after intravenous administration of A20.1Fc-Neurotensin and FC5Fc-Neurotensin to non-human primates.

		A20.1Fc-Neurotensin		FC5Fc-Neurotensin	
Parameter	Unit	Estimate	%CV	Estimate	%CV
C_max_	nmol/mL	9.4	4.2	8.0	6.2
t_1/2α_	h	3.7	13	3.3	16
t_1/2β_	h	61	7.5	67	10
CL	mL/h/kg	1.4	3.1	1.9	4.2
CL_D_	mL/h/kg	3.6	14	4.9	16
V_1_	mL/kg	33	4.2	38	6.2
V_2_	mL/kg	72	9.0	108	12
AUC_0–∞_	nmol × h/mL	85	3.1	65	4.2

Maximum predicted concentration (C_max_), Distribution half-life (t_1/2α_), Elimination half-life (t_1/2β_), Clearance (CL), Inter-compartmental Clearance (CL_D_), Volume of distribution of the central compartment (V_1_), and the peripheral compartment (V2), AUC from zero to infinity (AUC_0–∞_).

## Data Availability

The raw data supporting the conclusions of this article will be made available by the authors on request.
